# Endoscopic cardial constriction with band ligation in the treatment of refractory gastroesophageal reflux disease: a preliminary feasibility study

**DOI:** 10.1007/s00464-021-08397-y

**Published:** 2021-04-21

**Authors:** Zhi-Tong Li, Feng Ji, Xin-Wei Han, Rui Zhang, Li-Dong Chen, Chun-Xia Li, Li-Li Yuan, Zhong-Gao Wang, Kang-Dong Liu

**Affiliations:** 1 Department of Interventional Radiology, The First Affiliated Hospital, Zhengzhou University, No. 1, East Jian She Road, Zhengzhou, 450052 Henan Province People’s Republic of China; 2 Department of Respiratory and Critical Medicine, The First Affiliated Hospital, Zhengzhou University, No. 1, East Jian She Road, Zhengzhou, 450052 Henan Province People’s Republic of China; 3 Department of Gastroenterology, The First Affiliated Hospital, Zhengzhou University, No. 1, East Jian She Road, Zhengzhou, 450052 Henan Province People’s Republic of China; 4 School of Basic Medical Science, Zhengzhou University, Zhengzhou, 450001 Henan Province People’s Republic of China

**Keywords:** Gastroesophageal reflux disease, Extra-esophageal symptoms, Peroral endoscopic cardial constriction, Band ligation

## Abstract

**Background:**

Gastroesophageal reflux disease (GERD) is a common digestive disease, could cause extra-esophageal symptoms. Peroral endoscopic cardial constriction with band ligation (PECC-b) is a minimally invasive method for the treatment of GERD in recent years. The goals of this study were to evaluate the clinical efficacy of PECC-b to treat gastroesophageal reflux-related symptoms.

**Methods:**

A retrospective study of patients undergoing PECC-b between January 2017 and December 2018 at a single institution was conducted. All patients confirmed GERD by endoscopy, esophageal PH-impedance monitoring, esophageal manometry and symptom questionnaires. The outcome measures included reflux-related scores, patients’ satisfaction and drug independence after 12 months following surgery.

**Results:**

A total of 68 patients, with follow-up of 12 months post surgery, were included in the final analysis. The symptom scores were all significantly decreased as compared with preoperation (*P* < 0.05). The esophageal symptom scores showed a better improvement than extra-esophageal symptoms (*P* < 0.001). Fifty-three (77.9%) patients achieved complete drug therapy independence and 52 (76.5%) patients were completely or partially satisfied with the symptom relief following surgery.

**Conclusions:**

The PECC-b is a safe, effective and recommended approach for the control of GERD-related symptoms. Further multicenter prospective studies are required to confirm these outcomes.

**Supplementary Information:**

The online version contains supplementary material available at (10.1007/s00464-021-08397-y).

Gastroesophageal reflux disease (GERD) is one of the most common digestive diseases that could cause a series of symptoms and complications [1]. The most common symptoms are regurgitation or/and heartburn. GERD is believed to lead to extra-oesophageal symptoms and complications, primarily in the respiratory tract [2, 3], such as asthma, bronchitis, pneumonia, pharyngitis, snoring, obstructive sleep apnea. GERD also is found in 40% of patients with coronary artery disease confirmed by coronary angiography [4].

There are many treatments for GERD, including general lifestyle modification, medical therapy, endoscopic therapies, and laparoscopic antireflux surgery (LARS). When there is proton pump inhibitors (PPIs)-refractory GERD, complications of GERD or extra-esophageal manifestations, antireflux surgery is recommended. [5]. However, due to its invasiveness, high cost and postoperative complications, this approach is limited [6].

With the development of endoscopy, endoscopic treatment technology is a choice for the treatment of refractory GERD. The endoscopic Stretta procedure, anti-reflux mucosectomy (ARMS) and endoscopic fundoplication modalities are safe and effective modality and have been increasingly used [7–9]. However, several endoscopic treatments suffer from lack of feasibility, high costs and complications [8, 10]. In recent years, peroral endoscopic cardial constriction with band ligation (PECC-b) is a new, economical and easy to operate endoscopic technology for patients with typical GERD symptoms [11, 12]. However, it is unclear whether PECC-b is effective in patients with extra-esophageal symptoms.

This study was therefore conducted to evaluate results of the PECC-b procedure in patients with reflux-related symptoms.

## Materials and methods

### Study population

This was a retrospective study with consecutive cases enrolled from January 2017 to December 2018. The data for 68 patients with refractory GERD were reviewed and analyzed.

Clinical study was approved by the Ethics Committee of the First Affiliated Hospital of Zhengzhou University. Written informed consent for participation in the study was obtained from all patients.

The inclusion criteria were: (1) GERD diagnosed by endoscopic evidence of esophagitis or abnormal esophageal pH, a DeMeester score ≥ 14.72, and/or symptom correlation index ≥ 50% during 24-h PH-impedance monitoring; (2) lower than normal lower esophageal sphincter pressure detected by esophageal manometry; (3) non-hiatal hernia or small (< 2 cm) hiatal hernia.

The exclusion criteria included age < 18 years, previous esophageal or gastric surgery, coagulation disorders, connective tissue diseases, cardiac ulcer, esophageal stricture, impaired distal esophageal peristalsis, and/or autoimmune diseases.

### Surgical technique

**Fig. 1:**
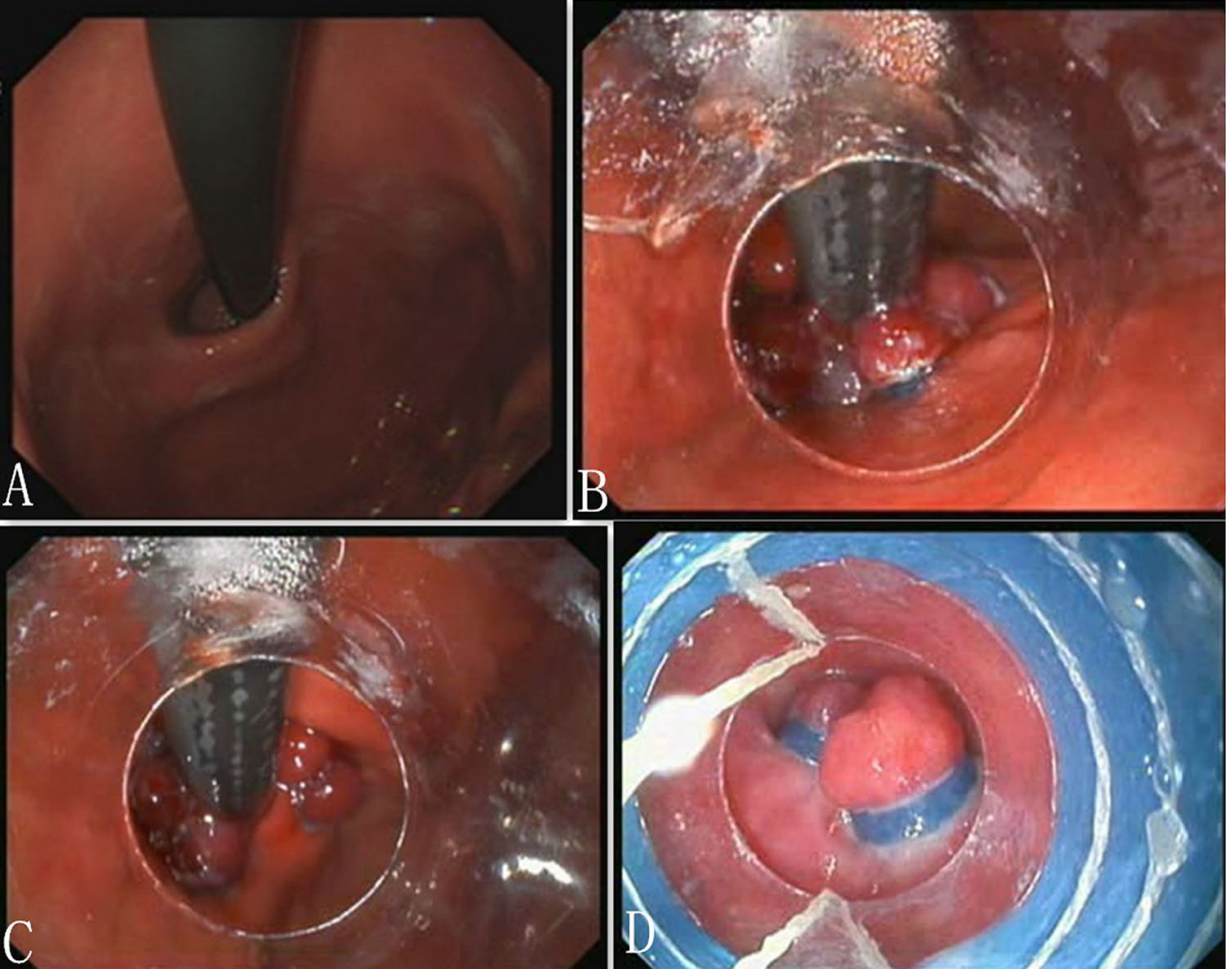
Procedure for cardial constriction with band ligation. **A** The cardia was endoscopically assessed in retroflex view. **B** Capture of mucosa one by one with the band ligation device at the level of the esophagogastric junction (EGJ), oriented towards the lesser curvature of the stomach. **C** Capture of mucosa one by one again under the first capture of mucosa to form two rows of ligation towards the lesser curvature. Don't capture mucosa on the side of the greater curvature. **D** Capture of mucosa with the band ligation device in the lower esophagus next to the EGJ

The surgical procedures used the principle of esophagogastric varices ligation. The endoscopic procedure was performed by an experienced endoscopist (L.D.C.) who had conducted more than 500 endoscopic treatment annually, 300 of them were endoscopic ligations and resections for esophagogastric varices and gastroesophageal neoplasia. Before procedures all patients were without diet and water for 6 h, the procedures were performed under intravenous fentanyl and midazolam with the continuous monitoring of vital signs. Patients were in left lateral decubitus position. The procedure was carried out using flexible endoscope of outer diameter 9.8 mm (GIFH 290; Olympus, Tokyo, Japan). Then the procedure was as follows (Fig. 1, Video 1):

Step 1: The endoscopist performed gastroscopy to observe the esophagus, stomach and duodenum. The esophagogastric junction (EGJ) was endoscopically assessed in retroflex view.

Step 2: A band ligation device (Speedband Superview Super 7; Boston Scientific Corporation, USA) was fitted on endoscope with large operating channel (3.8 mm). The mucosa was captured one by one with the band ligation at the level of EGJ towards the lesser curvature of the stomach. Capture of mucosa one by one again under the first capture of mucosa, forming two rows of ligation rings towards the lesser curvature of the stomach. The more mucosa was captured, the better. There were about 4–10 sets of ligation rings in the lesser curvature of the stomach. In order to avoid damage the angle of His, we did not use band ligature to narrow the cardia on the side of the greater curvature.

Step 3: Capture of mucosa with the band ligation device was scheduled along at the level of EGJ towards the esophagus. There were single-sided or double-sided ligation and about 1–3 sets of ligation rings in the esophagus..

The length of cardial constriction was about 4 cm length (3 cm in the lesser curvature and 1 cm in the esophagus). The number of ligation rings depended on the circumference of the cardia.

Drugs to prevent pain and bleeding were usually used on the day of surgery. Intravenous or oral omeprazole administration was used during postoperative hospitalization for about 3 days, and oral administration of 1000 mg of magnesium aluminum carbonate chewable tablets and 5 mg of mosapride three times a day for about 2 weeks. Eat liquid food on the first day after surgery, and eat soft food as much as possible within one month.

### Evaluation of outcome

The primary outcomes were comparisons of reflux-related scores, patients’ satisfaction and drug independence during postoperative follow-up. Postoperative follow-up was accomplished using outpatient visits or telephone.

The Reflux Diagnostic Questionnaire assessed gastroesophageal reflux-related symptoms scores [13]. The instrument used a six-point Likert scale system ranging from 0 to 5 to assess both the severity and frequency of heartburn, regurgitation, cough, wheezing, and chest pain as symptoms scores according to the Reflux Diagnostic Questionnaire (with revision) (Table 1) [14, 15]. More specifically, the frequency was graded as 0 (none), 1 (less than once per week), 2 (once or twice per week), 3 (three or four times per week), 4 (five or six times per week), and 5 (more than six times per week); the severity was graded as 0 (none), 1 (slight), 2 (mild), 3 (moderate), 4 (severe), and 5 (extremely severe). The total of the frequency scores and severity scores for each outcome measure was designated as the symptom score. Questionnaires were completed before the PECC-b treatment and then for 3, 6 and 12 months follow-up.

**Table 1:** Scoring method for the frequency and severity of GERD-related symptoms by using a Likert scale

Characteristics	Score
How often do you experience	
None	0
Less than once per week	1
Once or twice per week	2
Three or four times per week	3
Five or six times per week	4
More than six times per week	5
The severity degrees of GERD	
None	0
Slight	1
Mild	2
Moderate	3
Severe	4
Extremely severe	5

*GERD* Gastroesophageal reflux disease

According to the patients’ subjective feeling assessing medication independence and patients’ satisfaction, the effect was divided into four grades: “excellent” (complete resolution of symptoms), “good” (symptoms occurring once per month or less frequently), “fair” (symptoms occurring weekly or less frequently) and “poor” (symptoms occurring daily, or more often, or as severe as prior to treatment) [16].

**Fig. 2:**
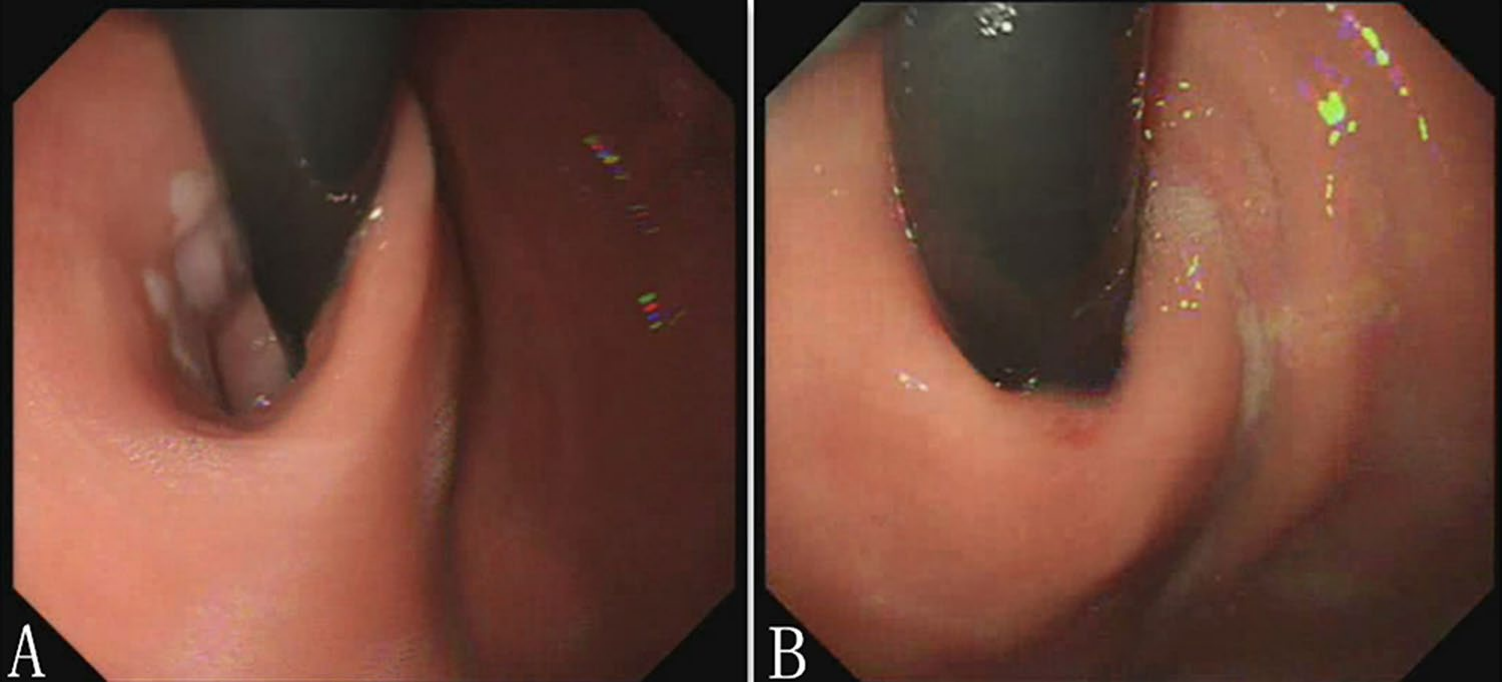
Gastroscopy showed the EGJ in retroflexion. **A** Retroflexed view of the EGJ preoperatively. **B** Retroflexed view of the EGJ 12 months postoperatively

The secondary outcome was follow-up endoscopy. The endoscopy was performed at 12 months to evaluate esophagitis and changes of EGJ (Fig. 2a and Fig. 2b).

#### Statistical analysis

Data analysis was performed using SPSS version 17.0 software (SPSS Inc, Chicago, IL, USA). Continuous variables were expressed as median or means ± standard deviations. Comparisons were made between preoperative and postoperative status using the Wilcoxon test or the *t* test as appropriate. All statistical tests were considered significant when two-tailed *P* values were less than 0.05.

### Results

#### Demographic findings and clinical symptoms

A total of 68 patients entered the study. The patient's baseline characteristics were summarized in Table 2. This study group consisted 38 men and 30 women, aged 18–73 (mean 45.8). Among them, 46 (67.6%) patients showed typical reflux symptoms (such as regurgitation, heartburn, bloating, and belching), 28 (41.2%) cases of cough and wheezing symptoms, 16 (23.5%) cases of rhinitis and pharyngitis symptoms, 10 (14.7%) cases of chest tightness and chest pain, 4 (5.9%) cases of salivation, 3 (4.4%) cases of snoring, 1 (1.5%) cases of laryngospasm.

**Table 2:** Demographic and clinical characteristics of the patients; n = 68

Characteristics	Value
Gender (Female/male), n	38/30
Age, year (range)	45.8 (18–73)
Symptom, n	
Gastrointestinal symptoms	46
Respiratory symptoms	28
Rhinitis symptoms	10
Pharyngitis symptoms	8
Coronary heart disease symptoms	10
Salivation	4
Snoring	3
Laryngospasm	1
Symptom duration, n	
≥ 1 year	46
< 1 year	22
Daily PPI use, n	56
Esophagitis (LA, A/B/C/D), n	30/12/0/0
Hiatal hernia	21
DMS, mean ± SD	44.1 ± 16.8
LESP, mmHg, mean ± SD	5.9 ± 6.2

*GERD* Gastroesophageal reflux disease, *HH* hiatal hernia, *LA* Los Angeles classification, *DMS* DeMeester score, *LESP* lower esophageal sphincter pressure (normal range: 13–43 mmHg)

#### Intra- and postoperative outcomes

All procedures were completed successfully by PECC-b methods. The mean operating time was 12.3 ± 3.2 min. Postoperatively, 25 patients with mild retrosternal pain and discomfort disappeared after 3 days. Twenty-eight patients had mild dysphagia after the procedure which did not require additional balloon dilation, then disappeared within 2 weeks. Others 10 patients had abdominal distension, 2 patients had mild hemoptysis and 1 patient had diarrhea, but these problems disappeared within 1–2 weeks. There were no serious complications during follow-up period.

#### Follow-up outcomes

**Fig. 3:**
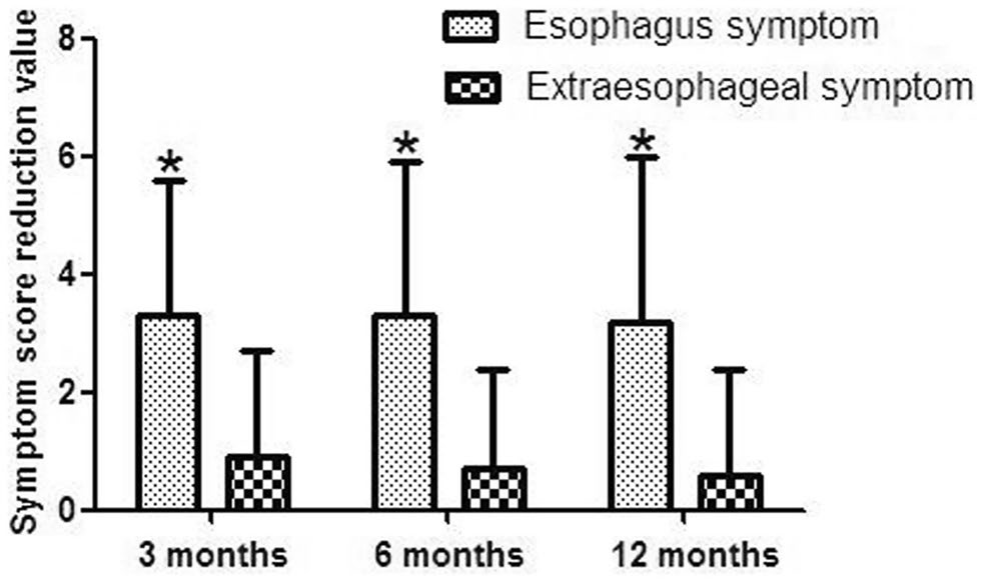
Effect of PECC-b treatment on the mean esophageal symptoms and extraesophageal symptoms. The esophageal symptoms resulted in significantly better outcomes than extraesophageal symptoms. *P < 0.05

At 3 months after surgery, the symptom scores were significantly lower than the score before the PECC-b procedure (all *P* < 0.05). As summarized in Table 3, the mean symptom scores of heartburn, regurgitation, cough, wheezing and chest pain were 4.51 ± 2.69, 4.46 ± 2.54, 2.06 ± 2.63, 1.92 ± 2.46 and 0.82 ± 1.91 before PECC-b treatment, and 1.12 ± 1.42, 1.21 ± 1.49, 0.78 ± 1.26, 0.82 ± 1.23 and 0.31 ± 0.72 after PECC-b treatment, respectively. By the end of the 1-year follow-up, the symptom scores also were decreased as compared with the corresponding values before the PECC-b procedure (*P* = 0, 0, 0, 0.004 and 0.018, respectively; Table 3). Furthermore, there were no significant change in symptom scores between 3, 6 and 12 months after PECC-b treatment (*P* > 0.05). Compared to the degree of decline in different symptoms, esophagus symptoms were significantly better with respect to extra-esophageal symptoms at 3, 6 and 12 months after PECC-b treatment (all *P* = 0; Fig. 3).

**Table 3:** Outcomes of PECC-b surgery with respect to symptom scores (n = 68)

Symptom	Preoperative score	Postoperative score 3 months	Postoperative score 6 months	Postoperative score 12 months	*P
Heartburn	4.51 ± 2.69	1.12 ± 1.42	1.19 ± 1.40	1.24 ± 1.52	< 0.05
Regurgitation	4.46 ± 2.54	1.21 ± 1.49	1.15 ± 1.59	1.28 ± 1.69	< 0.05
Cough	2.06 ± 2.63	0.78 ± 1.26	0.91 ± 1.34	0.99 ± 1.49	< 0.05
Wheezing	1.92 ± 2.46	0.82 ± 1.23	1.01 ± 1.42	1.22 ± 1.73	< 0.05
Chest pain	0.82 ± 1.91	0.31 ± 0.72	0.32 ± 0.80	0.41 ± 0.88	< 0.05

*Results of reflux-related symptom scores compared before and after PECC-b

The outcome of medication independence and patient's satisfaction also were showed at follow-up 12 months postoperatively. In the 28 patients who were using PPIs and montelukast sodium as needed complained of cough and wheezing symptoms before treatment, medication was completely eliminated in 18 (64.3%) of the patients. 35 (87.5%) of the other 40 patients were completely off PPIs. The total rate of medication independence was 77.9% (53/68). Out of the 68 patients, the outcomes for patient's satisfaction were reported as excellent in 28 patients (41.2%), good in 24 patients (35.3%), fair in 11 patients (16.2%) and poor in 5 patients (7.3%). In total, 52 out of the 68 (76.5%) patients were completely or partially satisfied with the symptom control.

Gastroscopy at 12 months was achieved in 28 (41.2%) patients with a pattern of narrowing of the EGJ scar (Fig. 2b). Esophagitis was documented in 28.57% (8/28) patients. Compared with the preoperative findings (42/68), the frequency of esophagitis was significantly lower after PECC-b procedure (*P* = 0.003).

### Discussion

PECC-b procedure for typical GERD symptoms has been confirmed to be effective. In this study, we not only investigated the effects of PECC-b treatment on typical GERD symptoms, but also evaluated extra-esophageal symptoms. We assessed incidence of complications, relative symptom improvement, patient's satisfaction and the frequency of esophagitis.

GERD is characterized by intermittent incompetence of the gastroesophageal junction, leading to the reflux of gastric contents into the esophagus, throat, mouth, even to the trachea. GERD has been shown to be associated with lung diseases such as asthma [17] and obstructive sleep apnea [18]. GERD also have recognized that laryngitis, cough, asthma, and dental erosion can be manifestations of the GERD syndrome, while sinusitis, pulmonary fibrosis, pharyngitis, recurrent otitis media may be associated with GERD [19]. In our study, 68 patients diagnosed with GERD were analyzed. Of these patients, 46 patients reported typical reflux symptoms, 28 cough and wheezing symptoms, 16 rhinitis and pharyngitis symptoms, 10 chest tightness and chest pain, and few other extra-esophageal symptoms.

The purpose of treating GERD is to maintain symptom relief and prevent complications. PPIs therapy involves an indefinitely prolonged or lifetime daily drug administration, which is associated with significant adverse effects [20]. Moreover, 40% of GERD patients are refractory to PPIs [21, 22]. PPIs do not improve asthma in patients with asymptomatic or silent reflux [23]. LARS is an important treatment method for medication-refractory GERD [24]. Anti-reflux surgery not only controls reflux symptoms, but also improves reflux-related extra-esophageal symptoms [15]. Previous studies have also reported 65%–75% respiratory symptom improvement in patients who had heartburn or regurgitation after fundoplication [25]. However, due to its side effects and complications, this approach is not very popular [6].

With the development of endoscopy, endoscopic antireflux technology has been increasingly used for PPIs-refractory GERD. Recently, PECC-b is a new, simple, easy and effective endoscopic technology for typical GERD symptoms [11, 12]. It is easy to be performed and needs just simple equipment (multi-ring ligator). The principle of PECC-b is similar to endoscopic varicose veins ligation. It also narrows the diameter of the cardia by rubber bands to prevent the reflux of gastrointestinal contents. In our PECC-b procedure, the ligation rings were placed at lesser curvature of the 3 cm-far of the EGJ and 1.0 cm above the EGJ along the esophagus. There were a total of 5–13 sets of ligation rings during procedure. So they could more effectively increase the lower esophageal sphincter pressure. In order to avoid damage the angle of His, we did not use band ligature to narrow the cardia on the side of the greater curvature, which was a factor involved in the prevention of GERD [26, 27]. These were different from the study of Hu et al. [12], which only two single-band ligation devices were placed at the greater curvature and lesser curvature, respectively. Compared to that used by Inoue et al. [8], our cardial constriction with band ligation procedure was simpler and easier to operate than ARMS because no mucosectomy area of EGJ was needed. Twenty-eight patients had mild dysphagia after the PECC-b procedure which did not require endoscopic dilation, and disappeared within 1–2 weeks, which was lower than after ARMS, as reported in the literature [8, 28].

In this study, we assessed whether the clinical efficiency of PECC-b for the treatment reflux-related symptoms. In previous studies, patients recorded a self-administered six-item diagnostic questionnaire GERD-Q [29, 30] before and after ARMS. The frequencies of six symptoms including heartburn, regurgitation, sleep disturbances due to reflux symptoms, the use of over-the-counter medications, epigastric pain, and nausea were evaluated with a 4-grade Likert scale (0–3). In this study, we used six-point Likert scale Reflux Diagnostic Questionnaire to assess the efficacy of PECC-b. The six-point scale was applied to assess the severity and frequency of heartburn, regurgitation, cough, wheezing, and chest pain as symptoms scores. The results showed that the symptom scores for regurgitation, heartburn, cough, wheezing and chest pain were significantly decreased during follow-up (Table 3). However, esophagus symptoms improved better with respect to extra-esophageal symptoms after PECC-b treatment (Fig. 3), which were the similar as in our study about Stretta procedure [14] and LARS [15].

We also assessed medication independence and patient's satisfaction with the treatment results. The outcome of medication independence and patient's satisfaction also were shown at follow-up 12 months postoperatively. 77.9% (53/68) patients were completely off medication. Fifty-two out of the 68 (76.5%) patients were completely or partially satisfied with the symptom control. A previous study had reported 69% and 54% of patients reported satisfaction with the management of their GERD symptom at 3 months and 6 months post-treatment, respectively [12]. Our results not only showed improved better reflux symptoms, but also extra-esophageal symptoms. The reason caused different efficacy might be due to different surgical procedures, which was similar as the effect of ARMS (GERD symptoms improved in 68% patients during a 2-year period) [28].

This was just a retrospective study. We did not use objective indicators for evaluation, such as esophageal pH and motility outcomes. However, objective evidence of reflux control was obtained by gastroscopic examination in some patients. Twenty-eight patients were available for gastroscopy at 12 months after surgery. Compared with the preoperative findings, the area of captured mucosa contracted, and the frequency of esophagitis was significantly lower after PECC-b procedure, which was similar as the effect of LARS [31].

However, this study has several limitations. First, the small number of enrolled patients prevents controls and double-blind analysis. Second, less evaluation measures and inconsistent standard procedures may make postoperative results less valuable. Finally, it is not clear whether PECC-b has long lasting effects.

In conclusion, PECC-b is a new, effective and safe method. It not only can control reflux symptoms, but also relieve reflux-related extra-esophageal symptoms. The postoperative results are stable and satisfactory. Multicenter randomized controlled study is required to confirm these findings.

## Supplementary Information

Below is the link to the electronic supplementary material.

Supplementary file1 (MP4 94,274KB)
